# Case Report: Profound Anemia in a 2-Year-Old Boy Associated with a Leech in the Oropharynx

**DOI:** 10.4269/ajtmh.23-0677

**Published:** 2024-04-02

**Authors:** Jerome Koleski, Amos Kipngetich Kurgat, Emilie Tindal, Jenna Haugen

**Affiliations:** ^1^ Department of Family Medicine, Kapsowar Mission Hospital, Kapsowar, Kenya;; ^2^ Department of Family and Community Medicine, University of Arizona, Tucson, Arizona;; ^3^ Department of Family Medicine, Kabarak University, Nakuru, Kenya;; ^4^ Department of Anesthesia, Kapsowar Mission Hospital, Kapsowar, Kenya;; ^5^ Faculty of Medicine, University of Southampton, Southampton, United Kingdom;; ^6^ Department of Family Medicine, Stanton Territorial Hospital, Yellowknife, NT, Canada

## Abstract

A 2-year-old boy presented to Kapsowar Mission Hospital in Kenya with a history of general tiredness associated with mild, unilateral epistaxis and one episode of hematemesis. On admission, he had a hemoglobin value of 3.5 g/dL, with a white cell count of 20.6 × 10^9^/L. The child was examined by the physician on call, with no source of bleeding found. Later that day, after a local physician noted that the presentation could be due to an unrecognized leech infestation, a deep examination of the oropharynx was performed with a laryngoscope and revealed a leech attached deep in the oropharynx. The anesthetist visualized the leech with a laryngoscope and removed it with Magill forceps. After the procedure and blood transfusion, the child’s hemoglobin level improved to 10.4 g/dL, and on the following day, the child was much improved in energy and was playing outside. He was discharged home on iron supplements and made a full recovery.

## INTRODUCTION

Leech infestation, although rare, should always be considered when a patient has unexplained anemia in a locale in which leech infestation is endemic.^1^ The literature from a number of middle-income countries reports leeches attaching themselves to various mucous membranes,^2^^,^^3^ including in the nasopharynx of adults^4^^,^^5^ and the oropharynx of children.^6^^,^^7^ The experience of our Kenyan coauthor is that the nasopharynx of a child is too small to house a full-sized leech, but the full-sized leech can fit in the nasopharynx of an adult.

This case report was determined by the University of Arizona Institutional Review Board to not involve human subjects as defined by Department of Health and Human Services and Food and Drug Administration regulations.

## CASE PRESENTATION

A 2-year, 10-month-old boy was brought by his mother to the Casualty Department at Kapsowar Mission Hospital (KMH) in Kapsowar, located in a remote area of northwest Kenya. The mother reported that the child had 2 days of tiredness and unilateral epistaxis, with the most recent epistaxis on the day of admission. There was one episode of hematemesis. The child had no decrease in oral intake or poor diet nor melena and/or bright-red rectal bleeding. There was no family history of bleeding diatheses, genetic bleeding disorders, or liver disease.

On admission, the child had a hemoglobin value of 3.4 g/dL. The white cell count was 20.6 × 10^9^/L, 76% neutrophils, 19% lymphocytes, 3% monocytes, 0% eosinophils, and 1% basophils. Malaria was not considered to be a likely cause as Kapsowar is not a malaria-endemic region and the elevation of the child’s village was above the elevation range of *Plasmodium falciparum*–carrying mosquitoes. Nonetheless, a malaria parasite test was performed and was negative. Of the limited tests available in the hospital laboratory, including the malaria parasite test, complete blood count, and peripheral smear, none of the test results provided any clue as to what might be causing such profound anemia with leukocytosis.

On examination, the child was listless and pale but had appropriate physical development for his stated age. Aside from marked pallor of the conjunctivae and mucous membranes, the child had normal physical findings. However, one of the local physicians pointed out that although the nasopharynx of children is too small to contain a full-sized leech, a leech could be present deep in the oropharynx.

**Figure 1.**
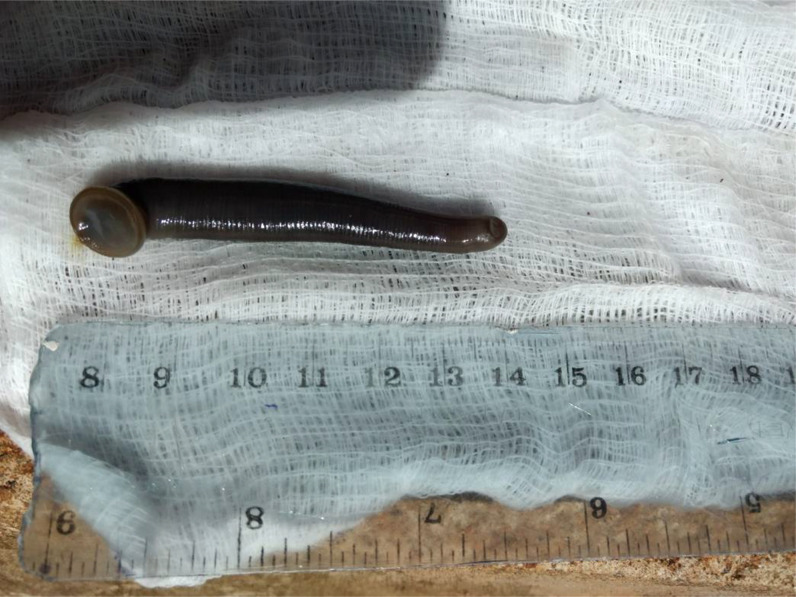
A 6-cm leech recovered from the boy’s oropharynx.

On deeper examination of the oropharynx, the tail of a leech was noticed, but it was too deep in the oropharynx to allow retrieval with forceps or hemostat. The child was taken to the operating theater, where the nurse anesthetist on duty examined the child with a laryngoscope, identified the leech, and removed the leech with McGill forceps (Figure 1).

Overnight, the child received two transfusions of 20 mL/kg whole blood and remained stable. In the morning after the leech was removed, the patient was playing normally. Because the child was at risk for other parasites, based on having already ingested water with a leech, and because of his geographic location in an arid region of Kenya, he was given empiric treatment with albendazole and metronidazole. He was discharged home on the day after leech removal with instructions to take an iron syrup.

## DISCUSSION

Leeches, scientific name *Hirudinea*, are blood-sucking parasites commonly found in freshwater sources. They are present on every continent but pose a particular problem to humans in areas where water sources are contaminated and where access to clean water is limited.^8^

The leech releases an anesthetic on attachment to the host, thus preventing the host from sensing the leech’s attachment. Leech saliva also contains hirudin, a potent anticoagulant. This results in prolonged bleeding from the leech bite, which can continue even after the leech stops feeding.^6^

There are three types of leeches: aquatic, marine, and terrestrial. Because of their weaker jaws, aquatic leeches, as occurred in this case, normally enter through body orifices and feed on mucosal surfaces of the upper aerodigestive tract (nasopharynx, larynx, esophagus, trachea, or even bronchi), lower genitourinary tract (urethra and vagina), and rarely, the eyes. The literature contains reports of leeches found in the nasopharynx of adults and older children,^2^^,^^9^^,^^10^ in the oropharynx of adults and children, in the vagina of premenarchal girls^11^ and postmenopausal women,^12^ and in patients varying in ages from 4 to 75 years. In addition, a leech was found in the urethra of a 13-year-old boy.^13^ The reaction of different medical professionals at our institution varied, based on their practice experiences in various geographic locations. Kenyan colleagues mostly responded, “Leeches are very common here, especially in the dry regions where there is poor access to safe drinking water.” North American colleagues’ reactions were varied, ranging from frank shock to “I never thought of that possibility.” However, the Canadian Medical Director of KMH said, “If you recall, I told you before you arrived that a child with unexplained anemia should be checked thoroughly for a leech.”

## CONCLUSION

Leeches can infest mucous membranes in any part of the body if contaminated water is ingested or used for bathing or swimming, with the infestation often occurring 2 weeks to 2 months prior to symptom presentation.^8^ Because the leeches attach when small and have anesthetic proteins, which make the patient unaware of the infestation, they can be easily missed in a routine physical exam. Extra care and, at times, extra equipment are needed to rule out this cause in cases of severe unexplained anemia.

In this case report, it was the knowledge of experienced expatriate physicians and Kenyan healthcare workers that enabled leech infestation to be considered as a possible diagnosis and the skill of the Kenyan nurse anesthetist in removing the leech that led to efficient treatment and recovery of the patient.

Unexplained, profound anemia, especially in children with limited access to clean water, should include leeches in the differential diagnosis. More thorough examination of the deep oropharynx to rule out a leech infestation should be done to avoid unnecessary referral and hematological workup.^4^
